# Born to Be a Risky Driver? The Relationship Between Cloninger’s Temperament and Character Traits and Risky Driving

**DOI:** 10.3389/fpsyg.2022.867396

**Published:** 2022-05-19

**Authors:** Timo Lajunen, Esma Gaygısız

**Affiliations:** ^1^Department of Psychology, Norwegian University of Science and Technology (NTNU), Trondheim, Norway; ^2^Department of Economics, Middle East Technical University, Ankara, Turkey

**Keywords:** risky driving, temperament, Cloninger, driver behaviour questionnaire, driver skill inventory, cooperativeness, persistence

## Abstract

Temperament refers to basic, largely inherited, relatively stable personality traits which have been present since early childhood. Considering the very fundamental role of temperament in human development and behaviour, it is reasonable to assume that temperament is also related to risky driving and drivers’ view of themselves as drivers. The aim of the present study was to investigate the relationships between Cloninger’s temperament dimensions, risky driving and drivers’ view of their perceptual motor and safety skills. The sample consisted of 335 Turkish drivers (aged 19–57; 53.7% men) who completed an Internet-based survey including Temperament and Character Inventory (TCI), Driver Behaviour Questionnaire (DBQ) and Driver Skill Inventory (DSI). Correlation analyses showed that TCI scale Cooperativeness correlated negatively with all DBQ scales indicating risky driving and positively with safety skills. In regression analyses after controlling age, gender and lifetime mileage, cooperativeness still was significantly related to all DBQ scales and safety skills. Persistence correlated negatively with ordinary violations, lapses and errors and positively with perceptual motor skills. In regression analyses, persistence was related to errors and lapses. Reward dependence was positively related to lapses and harm avoidance negatively to perceptual motor skills. The results of the present study indicate that largely innate temperament character traits may influence an individual’s predisposition to risky driving. Future studies about temperament and risky driving with larger samples allowing sub-group analyses are needed.

## Introduction

Since the conclusion by Greenwood and Woods (1919) that ‘…it seems that the genesis of multiple accidents under uniform external conditions is an affair of personality…’ (Greenwood and Woods, 1919), psychologists have been interested in individual differences in accident causation. While accident proneness theory, that is, the idea people who repeatedly have accidents are accident prone, has been widely refuted by theoretical and empirical grounds (McKenna, 1983; Salminen, 2001; Fay and Tissington, 2004; Purpura, 2019), the wide consensus among researchers seems to be that certain individual difference factors, such as cognitive factors or personality character traits, make some individuals more liable to accidents than others (McKenna, 1983; Lawton and Parker, 1998; Diamant and Rusou, 2021; Niranjan et al., 2022). One difference between the ‘differential accident involvement’ concept by McKenna (1983) and ‘accident proneness’ (Shaw and Sichel, 1971) is that the former refers to individual difference factors which increase the accident liability, whereas the latter focuses on the identification of ‘rotten apples’ who account for the most of the accidents. Nevertheless, we can safely conclude that certain cognitive performance-related and personality factors increase an individual’s risky behaviour and the likelihood of getting involved in a traffic accident.

The division to performance (i.e., driving skills) and behavioural (i.e., driving style) can be found in two widely used self-report measures of driving, namely, Driver Behaviour Questionnaire (DBQ; Reason et al., 1990) and Driver Skill Inventory (DSI; Lajunen and Summala, 1995). The DBQ asks drivers to report how often they have committed various aberrant driver behaviours during the past year. These aberrant behaviours were classified as errors, lapses, aggressive violations or ordinary violations. Errors are unwanted consequences of involuntary actions, whereas violations are based on conscious deviation from a rule or the safe practice. Errors can be further split into attention deficit related slips and memory failures (lapses), which both are results of cognitive processing problems, and, thus, related to driving skills and abilities. Ordinary violations do not have an aggressive motive while the main motive in aggressive violations is to show aggression (Lawton et al., 1997). While the DBQ reflects the division to driving skills and style in behaviour, the DSI measures the same distinction in terms of a driver’s self-assessed ‘strong and weak components’ in driving (Lajunen and Summala, 1995). The DSI items form two scales named perceptual motor skills (e.g., ‘fast reactions’ and ‘controlling the vehicle’) and safety skills (e.g., ‘driving carefully’ and ‘staying calm in irritating situations’). Although both scales are named as ‘skills’, they measure actually whether the drivers emphasise the vehicle handling or safety related behaviours when assessing their own driving. A review of earlier studies using the DBQ shows that the DBQ violations and errors correlate moderately with self-reported traffic accidents (de Winter et al., 2015). In addition, especially the DSI safety skills dimension has been reported to be related to risky driving and negative outcomes (Xu et al., 2018; Üzümcüoğlu et al., 2020; Lajunen and Özkan, 2021).

A quick search in Scopus database (30 January 2022) with search terms “(personality) AND (“driver behaviour” OR “driving behaviour”) yielded in 25 records for 2021, which shows that personality factors are still after hundred years of research seen as a relevant topic in traffic safety research. The personality factors, such as the Five-Factor Model (Costa and McCrae, 1992), have been used in numerous studies in relation to risky driving. Earlier studies about extraversion (Lajunen, 2001; Parr et al., 2016; Braitman and Braitman, 2017; Wang et al., 2018, 2020), neuroticism (Jovanović et al., 2011; Wang et al., 2018; Soori and Yousefinezhadi, 2020) and openness to experience (Parr et al., 2016; Wang et al., 2020) have shown that these personality factors often increase the propensity of various types of risky driving (e.g., distracted driving and aggression) while drivers scoring high in agreeableness and consciousness have been reported to commit risky driving less frequently (Jovanović et al., 2011; Wang et al., 2018, 2020; Hussain et al., 2020). When evaluating the effects of personality on risky driving, it is important to bear in mind that those relationships depend on the type of risky driving concerned: rule violations, driver aggression and distracted driving are related to specific personality factors or combinations of those factors.

When taking into account the vast number of studies about personality factors and risky driving, it is surprising how little interest temperament has raised among traffic researchers. Temperament refers to ‘basic, relatively stable personality traits which have been present since early childhood’ (Strelau, 2001). As the biological basis of personality (Goldsmith et al., 1987), temperament is determined by inborn neurobiochemical mechanisms and is, therefore, subject to slow changes caused by maturation and individual-specific genotype–environment interaction (Strelau, 2001). There seems to be a general consensus among temperament researchers that temperament forms the biologically based foundation of later-developing personality (Rothbart and Bates, 2006). Hence, temperament forms the core of personality dimensions on which different personality character traits develop. In this way, personality can be seen as a broader concept than temperament and the temperament traits as embedded into the broader personality factors (Shiner and DeYoung, 2013). Moreover, behaviour genetics research has demonstrated, for example, that each Five-Factor Model domain has a heritability of 40–50% similar to temperament factors (Jarnecke and South, 2017).

One of the widely used self-report instruments for measuring temperament is Cloninger’s Temperament and Character Inventory (TCI; Cloninger et al., 1994). The TCI is based on four genetically homogeneous and largely independently inherited temperament dimensions: novelty seeking (NS), harm avoidance (HA), reward dependence (RD) and persistence (PS). NS refers to a tendency towards exhilaration in response to novel stimuli; HA refers to a bias in the inhibition or cessation of behaviour; RD indicates a tendency to maintain or pursue ongoing behaviour: and PS refers to a predisposition to work hard to reach goals (Cloninger et al., 1993). In addition to these four dimensions of temperament, the TCI includes three character dimensions called self-directedness (SD), cooperativeness (C) and self-transcendence (ST), which reflect three aspects of self-concept (Cloninger et al., 1993). Individuals scoring high in SD are autonomous and show goal and value directed behaviours and ‘willpower’; a high score in C refers to acceptance, tolerance, empathy and helpfulness towards others; and ST refers to ‘identification with everything conceived as essential and consequential parts of a unified whole’ (Cloninger et al., 1993).

In terms of risk-taking, both the four temperament dimensions and three character traits can be expected to be related to risk-taking and self-view as a driver. One of the most impressive studies about the role of temperament in risky driving is the Australian Temperament Project (ATP), a large longitudinal community-based study, which has followed children’s psychosocial development from infancy to early adulthood. According to results, temperament style characterised by low task persistence/orientation was related to young adults’ risky driving (Vassallo et al., 2007, 2016). It should be noted; however, that temperament measurement was limited to the persistence/orientation factor, which is similar to Cloninger’s PS. Hence, the measurement of temperament was fairly narrow. In a study by Schwebel et al. (2007), correlations between Adult Temperament Questionnaire (Derryberry and Rothbart, 1988) scales and driver behaviour measured with the DBQ (Reason et al., 1990) were investigated among 101 elderly (≥ 75-year-old) drivers (Schwebel et al., 2007). Significant negative correlations were found between driver errors and all four temperament scales (activation control, attentional control, inhibitory control, high-intensity pleasure). Activation control and attentional control scores correlated (negatively) also with the DBQ lapses. Interestingly, temperament did not correlate with violations (Schwebel et al., 2007). It should be noted that these two studies were based on very different measures of temperament and risky driving. Also, the samples were very different from each other (young drivers, elderly), which might explain the different results.

Before focusing on any other individual differences, it should be noted that driving skills, behaviour and aversive outcomes, such as accidents and penalties, are strongly linked to driver’s gender, age and driving experience. A large body of earlier literature shows that men are riskier drivers than women and this difference is largest among young drivers (Evans, 2006; Shinar, 2017). Temperament character traits may interact with gender and age, because temperament can manifest in a different way among men and women and in different periods of life. For example, a young inexperienced male driver with high scores in Novelty Seeking might display extremely high traffic injury risk compared to a same aged woman scoring high in Novelty Seeking. Moreover, it can be assumed that older individuals seek sensations in a different manner than younger ones who are physically fit. In a very recent study among 18- to 25-year-old Finnish drivers, gender, age and driving experience were related to DBQ and DSI scale scores, which underlines the interaction between age, gender and mileage during the first 5 years of driving (Lajunen et al., 2022).

The aim of the present study was to investigate the relationships between the dimensions of Cloninger’s model (Cloninger et al., 1993; Cloninger, 1994) and driver errors, lapses, rule violations and aggressive violations (Reason et al., 1990). In addition to the direct relationships to driver behaviour, driver temperament might also influence drivers’ self-assessment as drivers. Therefore, the second aim of the study was to investigate the relationship between temperament and drivers’ self-evaluated driver skills and safety measured with DSI (Lajunen and Summala, 1995).

## Materials and Methods

### Participants

The data were collected by advertising the web link to SurveyMonkey among students at the Middle East Technical University, Ankara, Turkey. The students and their friends and relatives were invited to fill in an online survey. The online questionnaire contained information on the background to the study (driver behaviour), objectives (to study attitudes, opinions, interests and other personal feelings), voluntary nature of participation, declarations of anonymity and the confidentiality of all data. The instructions were the same as in the standard forms of the DBQ, DSI and TCI. The dataset consisted of 335 completed questionnaires. The mean age of drivers was 26.9 years (range: 19–57 years), and the standard deviation was 8.43 years. The mean lifetime mileage was 105,988 km (SD 960,766 km), and 53.7% were men.

### Materials

#### The Driver Behaviour Questionnaire

The Turkish translation of the 28-item DBQ (Özkan et al., 2006a) was used in the current study. The aberrant driver behaviours measured by the DBQ included ‘errors’ (8 items), ‘lapses’ (8 items), ‘ordinary violations’ (9 items) and ‘aggressive violations’ (3 items) scales. The self-reported behaviours in the previous year were recorded on a six-point Likert scale (1 = never, 6 = nearly all the time). The alpha reliability coefficients for the aggressive violations, ordinary violations, errors and lapses scales were 0.76, 0.91, 0.88 and 0.90, respectively.

#### The Driver Skill Inventory

The DSI is a 20 item self-reported measure of perceptual motor (11 items; e.g., fluent driving) and safety skills (9 items; e.g., conforming to the speed limits; Lajunen and Summala, 1995). The Turkish adaptation (Özkan et al., 2006b) was used in the present study. In the DSI, drivers are asked to rate how weak or strong they feel they were in given skills using a 5-point Likert scale (1 = very weak, 5 = very strong). The alpha reliability coefficients for the perceptual motor skills and safety skills scales were 0.94 and 0.86, respectively.

#### Temperament and Character Inventory

Temperament traits and character dimensions were measured with the TCI (Cloninger et al., 1994; Köse et al., 2004). The TCI contains 240 statements, which the respondents evaluate with ‘true’ and ‘false’ alternatives. The alpha reliability coefficients for the four temperament scales were 0.60 (NS), 0.70 (HA), 0.42 (RD), 0.40 (PS) and for the character dimensions 0.80 (SD), 0.79 (C) and 0.85 (ST). It should be noted that alphas for RD and PS were low, which may influence the results.

#### Demographic Measures

Respondents answered questions about their age, gender and their lifetime mileage.

### Statistical Analyses

Gender differences on the DBQ and DSI subscales were analysed using *t*-tests. Relationships between variables were analysed by using Pearson product–moment correlations and hierarchical regression analyses. All analyses were performed using SPSS (Statistical Package for Social Sciences) version 25.

## Results

### Descriptive Statistics and Gender Comparisons

Considering the vast differences between men and women in risky driving, men and women were compared by using *t*-test. Table 1 shows that men scored significantly higher in DBQ aggressive and ordinary violations, whereas no gender differences were found in lapses or errors. Similarly, men evaluated their perceptual motor skills to be higher than women. No difference was found in safety skills.

**Table 1:** Scale reliability coefficients as well as means (M) and standard deviations (SD) for men and women.

	*α*	Woman		Man		*t*-test
		*M*	SD	*M*	SD	
Aggressive violations	0.75	1.39	0.60	1.63	0.80	−5.55^*^
Ordinary violations	0.77	1.93	0.50	2.20	0.63	−7.75^*^
Lapses	0.62	2.01	0.46	1.85	0.45	5.20^*^
Errors	0.69	1.47	0.35	1.49	0.39	−0.88
Perceptual motor Skills	0.88	3.12	0.56	3.73	0.62	−16.55^*^
Safety Skills	0.82	3.56	0.57	3.31	0.64	6.70^*^

* *p < 0.001; df’s range in t-tests: 958–1,049*.

In terms of four temperament scales, men scored lower in NS, HA and RD, whereas no statistically significant difference was found in PS. When men and women were compared in terms of character dimension scores, women scored higher in SD and C than men, whereas no difference between gender was found in ST. Gender differences in scale scores might indicate that gender should be taken into account in further analyses.

### Pearson Product–Moment Correlations Between DBQ, DSI, and Temperament Scores

Correlations between driving-related variables (DBQ and DSI scales) and TCI scale scores are presented in Table 2.

**Table 2:** Pearson product–moment correlations between driving-related variables (DBQ and DSI scales), background variables (gender, age, mileage) and TCI scale scores.

	Aggressive violations (DBQ)	Ordinary violations (DBQ)	Lapses (DBQ)	Errors (DBQ)	Perceptual motor skills (DSI)	Safety skills (DSI)
Gender^1^	−0.19^***^	−0.28^***^	−0.01	−0.11	−0.39^***^	0.00
Age	−0.06	−0.07	−0.09	−0.10	0.12^*^	0.18^**^
Lifetime mileage (km)	−0.06	−0.04	−0.06	−0.05	−0.03	−0.04
Novelty seeking (NS)	0.01	0.07	0.11	0.07	0.03	−0.04
Harm avoidance (HA)	0.02	−0.01	0.12^*^	0.13^*^	−0.22^***^	−0.10
Reward dependence (RD)	−0.04	−0.05	0.04	−0.02	−0.08	0.10
Persistence (PS)	−0.12	−0.13^*^	−0.16^**^	−0.16^**^	0.13^*^	0.09
Self-directedness (SD)	−0.10	−0.15^*^	−0.13^*^	−0.20^***^	0.05	0.24^***^
Cooperativeness (C)	−0.24^***^	−0.28^***^	−0.21^***^	−0.27^***^	0.01	0.25^***^
Self-transcendence (ST)	−0.07	−0.13^*^	−0.14^*^	−0.09	0.02	−0.02

1 *man = 1; woman = 2*.

* *p < 0.05;*

** *p < 0.01; and*

*** *p < 0.001*.

Table 2 shows that both aggressive and ordinary violations correlated negatively with C while ordinary violations score correlated (although weakly) with PS and ST. In addition, both errors and lapses had statistically significant positive correlations with HA and significant negative correlations with PS, SD, C and ST. Perceptual motor skills correlated negatively with HA and PS, while safety skills correlated positively with SD and C.

Correlations between DSI and DBQ scores were also calculated. DSI perception motor skill scores correlated positively with aggressive violations (*r* = 0.13, *p* < 0.05) and negatively with lapses (*r* = −0.12, *p* < 0.05). Safety skills scores correlated negatively with aggressive violations (*r* = −0.20, *p* < 0.001) and ordinary violations (*r* = −0.24, *p* < 0.001) as well as with errors (*r* = −0.12, *p* < 0.05) and lapses (*r* = −0.16, *p* < 0.01).

While also the statistically significant correlations were generally weak (range: 0.12–0.28), these correlations show that temperament character traits are related to risky driving and drivers’ view of himself/herself as a driver.

### Temperament and Risky Driving: Regression Analyses

Correlations presented in Table 2 show that several TCI scales correlated with risky driving measured with the DBQ. The correlation results, as well as the comparisons between genders, show that male gender correlated with risky driving, namely, with violations and perceptual motor skills. To find out the significant temperament dimensions and character predictors of risky driving, four multiple regression analyses were performed (one for each DBQ scale). In the first step, background variables age, gender and lifetime mileage were entered into the model. In the second step, seven TCI scale scores were entered into the model by using the stepwise method (criterion to enter: *p* < 0.05). In the third step, moderation terms (standardised temperament score × gender) were entered into the model by using stepwise selection.

Table 3 shows the results of four regression analyses (the last step results) in which DBQ scale scores (aggressive violations, ordinary violations, errors and lapses) were predicted by the TCI temperament and character scales. In each analysis, the effect of mileage, gender and age were controlled.

**Table 3:** Temperament and risky driving: regression analysis results for DBQ variables as the dependent variable.

	*B*	Beta	*t*	95.0% CI Lower bound	95.0% CI Upper bound
Aggressive violations (*r*^2^ = 0.11)					
Age	−0.01	−0.05	−0.76	−0.02	0.01
Gender	−0.35	−0.17	−2.80^**^	−0.60	−0.10
Lifetime mileage	0.00	−0.02	−0.42	0.00	0.00
Cooperativeness (C)	−0.04	−0.22	−3.59^***^	−0.06	−0.02
Novelty seeking × gender	0.15	0.14	2.44^*^	0.03	0.27
Ordinary violations (*r*^2^ = 0.14)					
Age	−0.01	−0.05	−0.91	−0.02	0.01
Gender	−0.44	−0.24	−4.19^***^	−0.65	−0.23
Lifetime mileage	0.00	−0.01	−0.13	0.00	0.00
Cooperativeness (C)	−0.04	−0.23	−3.88^***^	−0.05	−0.02
Lapses (*r*^2^ = 0.09)					
Age	0.00	−0.02	−0.33	−0.01	0.01
Gender	0.04	0.02	0.40	−0.15	0.23
Lifetime mileage	0.00	−0.03	−0.59	0.00	0.00
Cooperativeness (C)	−0.03	−0.25	−3.86^***^	−0.05	−0.02
Persistence (PS)	−0.07	−0.14	−2.38^*^	−0.12	−0.01
Reward dependence (RD)	0.04	0.14	2.14^*^	0.00	0.07
Errors (*r*^2^ = 0.10)					
Age	0.00	−0.03	−0.57	−0.02	0.01
Gender	−0.09	−0.05	−0.92	−0.29	0.11
Lifetime mileage	0.00	−0.02	−0.32	0.00	0.00
Cooperativeness (C)	−0.04	−0.25	−4.13^***^	−0.05	−0.02
Persistence (PS)	−0.07	−0.14	−2.36^*^	−0.13	−0.01

* *p ≤ 0.05;*

** *p ≤ 0.01; and*

*** *p ≤ 0.001*.

Table 3 shows that the most important TCI scale in terms of risky driving was cooperativeness (C), which was negatively related to every DBQ scale. The second most important temperament dimension was persistence (PS) which was negatively related to lapses and errors but not with violations. In addition, reward dependence predicted lapses but not the other DBQ scale scores.

In general, gender did not seem to moderate the relationships between temperament and risky driving. The only moderation effect of gender was found for novelty seeking and aggressive violations relationship: among women, novelty seeking was positively related to aggressive violations (*r* = 0.16, *p* = 0.08), whereas the relationship was negative among men (*r* = −0.09, *p* = 0.27). It should be noted, however, that these results were statistically non-significant.

**Figure 1:**
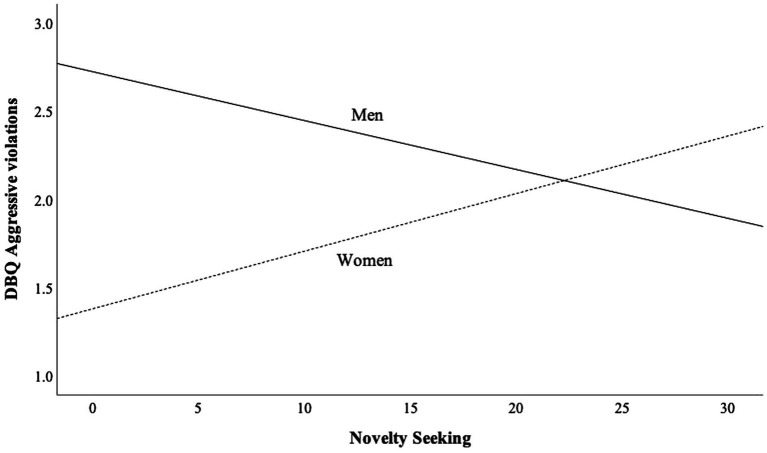
Moderation effect of gender on the relationship between novelty seeking (NS) and aggressive violations (DBQ).

Figure 1 show the moderation effect of gender on the relationship between novelty seeking and aggressive violations. Among men, the aggressive violation score seems to decrease as function of novelty seeking whereas the opposite occurs among women. In low levels of novelty seeking, the difference between men and women is highest while in the highest levels of novelty seeking women seem to score higher than men. It should be noted, however, that the correlation between aggressive violations and novelty seeking was statistically non-significant for both men (*r* = −0.09, *p* = 0.272) and women (*r* = 0.16, *p* = 0.075). Hence, the results related to moderation should be seen as tentative.

### Temperament and Self-Evaluated Perceptual Motor and Safety Skills: Regression Analyses

Table 2 shows that TCI scales HA, PS, SD and C correlated with the DSI scales. Also, gender correlated moderately with perceptual motor skills (*r* = −0.39) and age correlated positively with perceptual motor skills (*r* = 0.12) and safety skills (*r* = 0.18). Therefore, in regression analyses, gender, age and mileage were entered to model at the first step. In the second step, seven TCI scale scores were entered into the model by using the stepwise method (criterion to enter: *p* < 0.05). In the third step, moderation terms (standardised temperament score × gender) were entered into the model by using stepwise selection.

Results of the two regressions analyses are presented in Table 4. In the first model, gender and harm avoidance predicted (negatively) perceptual motor skills. In the second model, safety skills were predicted by cooperativeness. The only significant moderator term was the novelty seeking × gender, which had a negative relationship to safety skills. Novelty seeking was negatively related to safety skills among women (*r* = −0.14, *p* = 0.13) and positively among men (*r* = 0.06, *p* = 0.48). However, both relationships were statistically non-significant.

**Table 4:** Temperament and self-assessed driving skills: regression analysis results for DSI variables as the dependent variable.

	*B*	Beta	*t*	95.0% CI Lower bound	95.0% CI Upper bound
Perceptual motor skills (*r*^2^ = 0.19)					
Age	0.01	0.09	1.55	0.00	0.02
Gender	−0.58	−0.36	−6.38^***^	−0.75	−0.40
Lifetime mileage	0.00	−0.02	−0.38	0.00	0.00
Harm avoidance (HA)	−0.03	−0.16	−2.93^**^	−0.04	−0.01
Safety skills (*r*^2^ = 0.10)					
Age	0.01	0.13	2.11^*^	0.00	0.02
Gender	−0.02	−0.02	−0.27	−0.20	0.16
Lifetime mileage	0.00	−0.08	−1.35	0.00	0.00
Cooperativeness (C)	0.03	0.24	3.95^***^	0.02	0.05
Novelty seeking × gender	−0.10	−0.13	−2.21^*^	−0.19	−0.01

* *p ≤ 0.05;*

** *p ≤ 0.01; and*

*** *p ≤ 0.001*.

**Figure 2:**
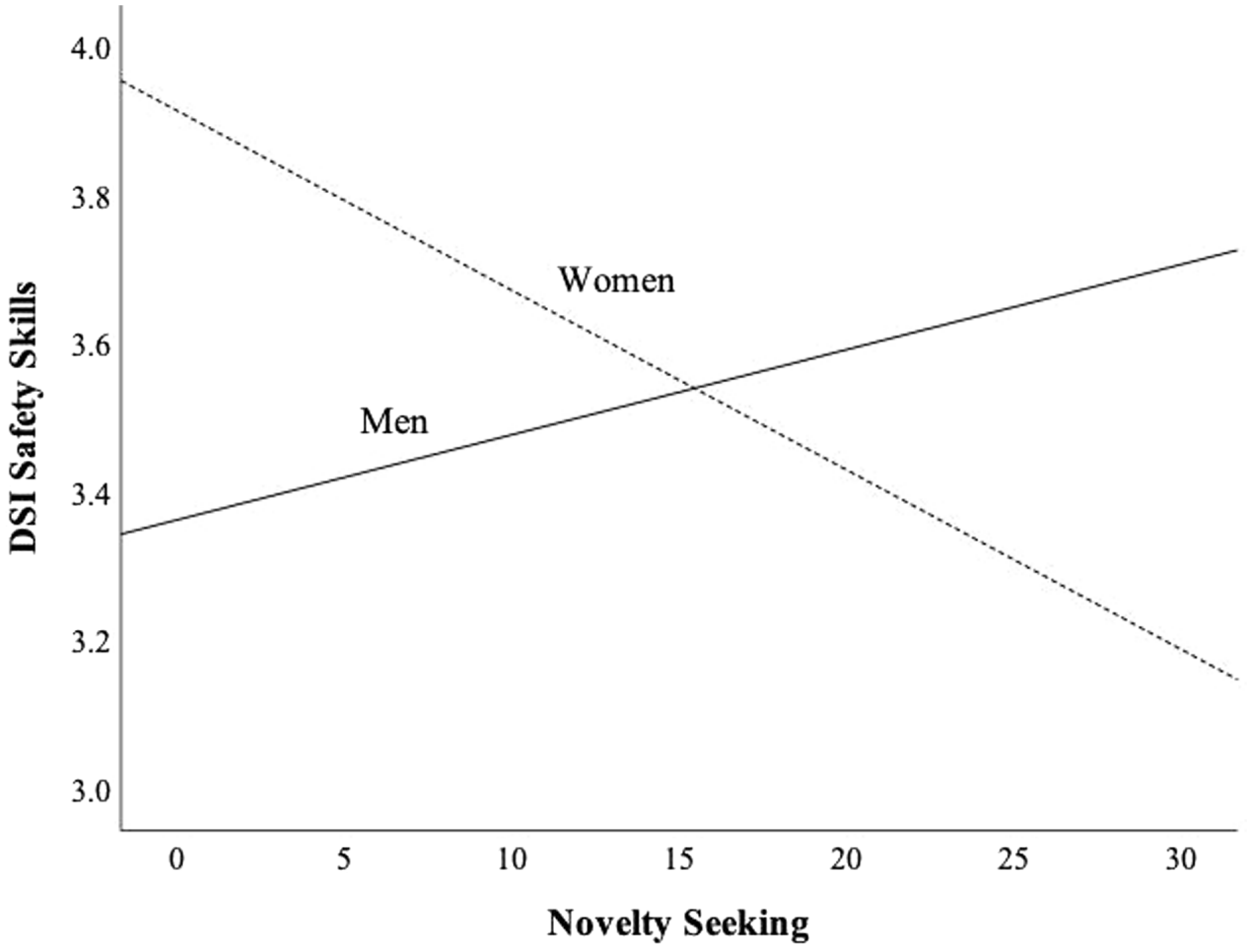
Moderation effect of gender on the relationship between novelty seeking (NS) and safety skills (DSI).

Figure 2 shows the moderation effect of gender on the relationship between novelty seeking and safety skills. Among women, the safety skills score seems to decrease as function of novelty seeking whereas the opposite occurs among men. In low levels of novelty seeking. In low level of novelty seeking women score higher than men in safety skills whereas in high levels of novelty seeking men score higher in safety skills than women. Men and women seem to be in the same level of safety skills when novelty seeking score is around the score 15. It should be noted, however, that the correlation between safety skills and novelty seeking was statistically non-significant for both men (*r* = −0.06, *p* = 0.471) and women (*r* = −0.14, *p* = 0.128). Hence, the results related to moderation should be seen as tentative.

## Discussion

Temperament can be defined as individual differences which emerge very early in life, are largely heritable and determined inborn neurobiochemical mechanisms linked to emotionality and arousability (Goldsmith et al., 1987; Strelau, 2001). When taking into account the very fundamental role of temperament in human development and behaviour as well as its close relationship to personality factors, it is reasonable to assume that temperament is also related to risky driving and drivers’ view of themselves as drivers. In the present study, the temperament and character dimensions of Cloninger’s model were studied in relation to aberrant driver behaviour—errors and violations—and drivers’ self-assessment of their perceptual motor skills and safety skills. A large body of literature shows that DBQ correlates with at least self-reported accidents (De Winter and Dodou, 2010; af-Wåhlberg et al., 2011; de Winter et al., 2015). Similarly, drivers’ self-assessment of their skills has been found to be related to self-reported accidents and penalties (Lajunen et al., 1998; Özkan et al., 2006b; Warner et al., 2013; Liu et al., 2021).

While the personality has been studied in relation to driver behaviour since the famous statement ‘a man drives as he lives’ by Tillman and Hobbs (1949, p. 329), temperament has attracted almost no attention at all. This is surprising, taking into account the biological and hereditary basis of temperament, which is also seen in personality factors. As the Australian Temperament Project (ATP) shows, temperament style measured in early childhood predicts risky driving as a young adult (Vassallo et al., 2007, 2016). In the present study, four of Cloninger’s TCI scales (PS, SD, C, ST) correlated negatively with lapses and three scales (PS, SD, C) correlated negatively with errors, which is in line with results by Schwebel et al. (2007), who used Adult Temperament Questionnaire (ATQ) for measuring temperament. In Schwebel et al. (2007), ATQ temperament scales had non-significant correlations to violations, which can be partly be explained by a small sample size (*n* = 101) or the sample including only elderly drivers, whose inclination to violations is anyway lower than among young or middle-aged drivers. Interestingly, temperament dimension ‘harm avoidance’ correlated positively with errors and lapses but negatively with perceptual motor skills, which is somewhat surprising. Harm avoidance might lead to low trust on one’s vehicle handling skills, which in turn would make decisions while driving uncertain and, thus, increase the number of lapses and errors. Lapses and errors are the result of inadequate or slow cognitive processing and harm avoidance might make those processes less efficient and lead to difficulties in rapid decision making while driving. For example, a driver scoring high in harm avoidance and thus excessively avoiding risks might hesitate too long to overtake a slow vehicle in front and miss the optimal moment for overtaking. Since driving is an optimisation task, both too hasty and postponed decisions increase the risk of errors.

In the present study among Turkish drivers aged between 19 and 57, aggressive violations correlated significantly (negatively) with the character trait ‘cooperativeness’ and ordinary violations also correlated negatively with temperament trait ‘persistence’ and character trait ‘self-directedness’. In regression analyses, especially cooperativeness appeared as an important factor together with persistence and reward dependence. Cooperativeness was also related to the DSI safety skills. These results show that both Cloninger’s temperament traits and character dimensions influence risky driving and drivers’ view of themselves. Obviously, drivers reflect both their inherited temperament traits, such as persistence, and learned character dimensions, such as cooperativeness in their driving behaviour. In future, more research is needed for distinguishing the effects of inherited traits and learned character traits in risky driving.

It is easy to understand why cooperativeness appeared as an important characteristic in both correlations and regressions analyses. A cooperative person is helpful and unselfish and avoids competition (Cloninger et al., 1993; Cloninger, 1994). The traffic system is based on collaboration among road users and on respect for rules and each other. A person scoring high on the collaboration can be expected to have a friendly attitude to other people, in this case, other road users, avoiding competition and being tolerant to others’ mistakes. These are the character traits that not surprisingly reduce the likelihood of aggressive and rule violations as well as errors due to driver distraction. Persistence refers to a person’s tendency to work hard to achieve the desired goal and inclination not to give up (Cloninger et al., 1994). Persistence can be especially important when learning to drive. In the present study, persistence correlated with the self-assessed perceptual motor skills, which supports the idea that persistence as temperament dimension might be related to one’s tendency to set himself/herself high goals as a driver. Reward dependence as temperament trait did not appear as on important factor in this study. This is not surprising, because this study focused on aberrant driver behaviours, which very seldom lead to reward or punishment, such as social approval or disapproval. RD might be more relevant in positive driver behaviours (Lajunen and Özkan, 2005) in which social reward is more likely (e.g., giving way to a pedestrian and being thanked). Future studies about temperament, personality and driving should include more driver behaviours than only aberrant behaviours.

The present study has some limitations, which should be taken into account when evaluating the results. While Cloninger’s temperament dimensions and character traits correlated with risky driving and self-assessed skills, the correlations were relatively weak. While strong or even moderate correlations between individual difference factors and risky driving are in general rate in literature, the weak correlations reported in this study might be related to the sample, which was too small for sub-group analyses. It can be expected that temperament as a strongly biological standing has a very different role among different age groups and genders. This study was conducted in Turkey and might partly reflect Turkish cultural gender roles in driving but also in expression of character traits. Cross-cultural studies would be very useful to identify cultural components in driving and temperament. In future studies, much larger samples from different countries are needed for sub-group analyses. Second, risky driving was measured with a self-report instrument. As described by Lajunen and Özkan (2011), self-reports of driver behaviour have certain shortcomings (e.g., social desirability bias; Lajunen and Özkan, 2011). While self-reports, such as TCI, are a handy way to measure temperament traits and character types, other ways of measuring temperament and personality, such as peer ratings, could be used even while driving. For example, spouses of the drivers have observed their partners while driving and in other non-driving-related situations. In future research, driver temperament and personality character traits as well as driving style could be assessed by using these ‘partner ratings’ in addition to self-reports. In personality research in driving context, this kind of designs are unfortunately rarely used. Finally, the sample was collected by using ads at the university. This kind of sampling is far from representative. While the participants answered anonymously in Internet and did not gain anything from participating, the sample does not represent the Turkish population, which should be considered when evaluating the results. On the other hand, it is almost impossible to collect representative samples in Turkey by using postal surveys or household interviews. The response rate among university students and their family members and friends is much higher than in randomly distributed surveys.

In conclusion, the current study shows that temperament traits and character traits should be taken into account when investigating individual differences in risky driving. Especially cooperativeness and persistence seem to be promising factors related to risky driving.

## Data Availability Statement

The raw data supporting the conclusions of this article will be made available by the authors, without undue reservation.

## Ethics Statement

Ethical review and approval was not required for the study on human participants in accordance with the local legislation and institutional requirements. Written informed consent for participation was not required for this study in accordance with the national legislation and the institutional requirements.

## Author Contributions

TL and EG contributed equally in every stage of the study, including planning, data collection, analyses, and preparation of the manuscript. All authors contributed to the article and approved the submitted version.

## Conflict of Interest

The authors declare that the research was conducted in the absence of any commercial or financial relationships that could be construed as a potential conflict of interest.

## Publisher’s Note

All claims expressed in this article are solely those of the authors and do not necessarily represent those of their affiliated organizations, or those of the publisher, the editors and the reviewers. Any product that may be evaluated in this article, or claim that may be made by its manufacturer, is not guaranteed or endorsed by the publisher.
